# Longitudinal Patterns of Suicidality Among Heroin Users in Johannesburg, South Africa: A Need for Suicide Screening and Intervention

**DOI:** 10.3389/fpsyt.2022.883878

**Published:** 2022-05-31

**Authors:** Nirvana Morgan, Ellen-ge Denton, Ugasvaree Subramaney, William Daniels, Tilman Steinert

**Affiliations:** ^1^Department of Psychiatry, Ulm University, Ulm, Germany; ^2^Department of Psychology, College of Staten Island, City University of New York, New York, NY, United States; ^3^Department of Psychiatry, School of Clinical Medicine, University of the Witwatersrand, Johannesburg, South Africa; ^4^School of Physiology, University of the Witwatersrand, Johannesburg, South Africa

**Keywords:** suicide, heroin, nyaope, screening, brief interventions, low-and middle-income countries, detoxification

## Abstract

**Objectives:**

The objective of the study was to longitudinally assess the outcomes and correlates of suicidal ideation and behavior (SIB) among heroin users who attended inpatient detoxification and psychosocial rehabilitation. SIB was assessed in 300 heroin users upon entry into inpatient detoxification (baseline) as well as 3-months (t1) (*n* = 252; 84%) and 9-months (t2) (*n* = 225; 75%) post treatment. Multivariable logistic regression was used to determine the demographic, clinical and treatment related factors that increased the risk for a high SIB score.

**Results:**

From baseline to t1 there was a significant decrease in the proportion of those who endorsed SIB (68.7 vs. 38.9%, *p* < 0.0001). There was an increase in the proportion of those who endorsed SIB from t1 to t2 (38,9 vs. 47.1%, *p* = 0.047). There was a significant increase in the proportion of those reporting suicide likely in the near future from baseline to t1 (8.7 vs. 16.3%: *p* < 0.0049) and this was repeated from t1 to t2 (22.7%) (t1 vs. t2: *p* = 0.031). After controlling for all other variables, a comorbid mental illness (MI) at baseline was a significant independent risk factor for a high SIB score at t1(RR 1.63; 95% CL 1.30–2.03) (*p* < 0.0001) and a comorbid MI at t1 increased the risk for a high SIB score at t2 (RR 2.73; 95% CL 1.78–4.19) (*p* < 0.0001). A poorer general health score and poorer social functioning score at baseline were associated with a high SIB score at baseline (RR 1.02; 95% CL 1.01–1.04) (*p* = 0.001) and t2, respectively (RR 1.07; 95% CL 1.04–1.11) (*p* < 0.0001).

**Conclusions:**

Among heroin users, a comorbid mental illness, poorer physical health and poorer social functioning are important factors to consider in suicide risk assessment. Although there were decreases in overall SIB 3 months after detoxification, this trend was not sustained at 9-month follow-up. After detoxification there were significant increases in the proportion of those reporting a likelihood of suicide in the following 3 months. The results suggests that the treatment exposure did not adequately mitigate suicide risk. There is a need for review of the treatment as well as targeted screening and management of SIB in heroin users attending treatment services.

## Introduction

The most common causes of death among heroin users are drug overdose, medical comorbidities, trauma, and suicide (1). Three to 35% of deaths can be attributed to suicide among heroin users (1–6). In fact, death due to suicide had the highest^1^ standardized mortality ratio (SMR) for all-cause mortality among heroin users in Taiwan (7). Clinical treatment is challenged to manage these patients' chronic substance use symptoms comorbid with ongoing suicide risk. It is estimated that the lifetime prevalence of suicide attempts ranges from 17 to 40% and suicidal ideation^2^ between 52 and 60% for heroin users (8). Several psychiatric, general medical and social risk factors which are known to increase risk for suicide in the general population have particular salience in heroin users and may account for high rates of completed suicide as well as suicidal ideation and behavior (SIB) (1). Despite the high risk for suicide among heroin users, there is a paucity of longitudinal data on SIB in heroin users who have attended treatment facilities. Disentangling these factors, over time, is vital to improved understanding of substance-related rehabilitative treatment, suicide risk, and treatment outcomes.

Three longitudinal studies from high income regions, namely Australia (9), United States (10), and the United Kingdom (11), report on the changes in prevalence of SIB in heroin users who received treatment. The treatment modalities examined were outpatient opioid agonist maintenance treatment (OAMT), short-term inpatient treatment, and residential rehabilitation. In these studies, at baseline, 22.5–33% endorsed SIB. There was a trend toward significantly decreasing prevalence of SIB (6.7–15.4%) at 1 year (9, 10) and 2 year (11) follow-up. These data suggest that within these countries, the incidence of SIB decreases with treatment services offered to heroin users. However, these data cannot be extrapolated to SIB outcomes in low- and middle-income country (LAMIC) settings where healthcare systems tend to be fragmented, under-resourced and where OAMT is not always standardly offered to heroin users (12–15). Further, treatment approaches and interventions for suicide in heroin users require consideration of specific sequela for SIB and a wide range of ongoing risk among heroin users.

Compared to risk for completed suicide, risk factors for attempted suicide in heroin users are more frequently analyzed and can be grouped into the following broad categories: gender, psychopathological symptoms, family dysfunction, social dysfunction and drug dependence. As is the trend with gender and suicide in the general population, men who use heroin have higher rates of completed suicide than women, however women who use heroin have higher rates of suicide attempts (1). With regard to psychopathological symptoms, a diagnosis of major depression, depressive symptoms, anxiety symptoms, post-traumatic stress disorder and antisocial personality disorder have been reported to increase the risk for suicide attempts, and in some instances completed suicide in heroin users (6–8, 16–19). Reports on suicide decedents in the general population show that one-third of people who died by suicide had a general medical illness at the time of death (20) and people with HIV have approximately a seven times higher risk for suicide than the general population (21). Although infrequently evaluated, comorbid general medical illness as a risk for SIB among heroin users has been previously reported (17, 22).

Mental and physical health comorbidities are complicated by social risk factors for suicide in heroin users. Specifically, poor social functioning (23–26), absent parents during childhood, parental psychopathology and parental drug and alcohol problems contribute to suicide risk in heroin users (1, 27). Substance use-associated factors such as the severity of heroin addiction, length of heroin use, needle sharing and polysubstance use also contribute to increased risk for suicide attempts (18, 28, 29). Most of the risk factors for suicide attempts in heroin users parallel the risks reported in the general population. However, compared to the general population, factors such as social dysfunction, mental illness, polysubstance use, and physical illness occur at much higher rates in heroin users (1).

Given the complex constellation of risk factors for suicide among heroin users and considering that 75% of suicides occur in LAMIC, it is imperative to better understand the patterns and risks for SIB among heroin users in LAMICs (30). Moreover, in order to determine the impact of detoxification-based treatment on SIB in this chronically at-risk group, longitudinal analyses of suicide risk factors from these settings are of particular importance. Therefore, the objective of the study was to longitudinally assess the outcomes and correlates of SIB among heroin users who attended inpatient detoxification-based treatment facilities in Johannesburg, South Africa.

## Methods

### Setting

Between 2017 and 2018, heroin users from two inpatient drug and alcohol rehabilitation facilities in the greater Johannesburg area of South Africa were screened and enrolled into the study. Both facilities were state-funded. They offered 1 week of medically supported detoxification followed by 6 to 8 weeks of mostly group-based psychosocial therapy, which was offered by social workers and addiction counselors. Inpatient detoxification and psychosocial therapy are the standard and predominant treatment modalities offered to heroin users in the greater Johannesburg region of South Africa. Methadone was prescribed for patients only during detoxification (1 week) as opioid agonist maintenance therapy (OAMT) and was not part of the treatment protocol at the rehabilitation facilities. One of the facilities had 20 beds and was exclusively for men and the other had 200 beds of which 20 were reserved for women. Upon leaving rehabilitation, the majority of patients returned to their homes and were encouraged to seek further outpatient treatment at a community-based social service center. The treatment available at the community-based facilities consisted primarily of individual therapy with a social worker, group therapy and Narcotics Anonymous meetings. OAMT is not standardly offered at substance rehabilitation treatment facilities in the greater Johannesburg region of South Africa.

### Procedure

Newly admitted patients were screened for study inclusion (enrollment). Inclusion criteria were met if the potential participant was using heroin (called nyaope or thai in the Gauteng province of South Africa) in the 6 months prior to admission, was older than 18 years, had capacity and willingness to sign informed consent and could provide locator information for follow-up. Potential participants who were assessed as medically unstable due to withdrawal symptoms or active symptoms of severe mental illness (such as psychosis or mania) were not eligible to participate in the study. Participants who had attempted suicide during their admission to rehabilitation or described plans and intent for suicide in the ensuing 3 months were excluded from the study. Participants were interviewed within their first 3 weeks of admission to rehabilitation (baseline) and then 3-months (t1) and 9-months (t2) after they left rehabilitation. All interviews were conducted face-to-face by the principal investigator (NM), who is a registered psychiatrist, but was not a staff member at either of the facilities. At both follow-up points a HOMEMED 6-panel Multi-Drug Urine Test (MDUT) was administered to participants who were able to provide a sample. The MDUT screened for tetrahydrocannabinol, opioids, cocaine, amphetamine, methamphetamine and amphetamine. MDUTs were done on 196 (76.6%) of participants at 3 months and 199 (88.4%) at 9 months. The concordance between self-report and MDUT (where data were available for both) was 65% at 3 months and 71% at 9 months. The study was reviewed and approved by the University of the Witwatersrand Human Research Ethics Committee (M1704100). Study data were collected and managed using REDCap electronic data capture tools hosted at the University of the Witwatersrand (31). Further specifics of the study procedure are detailed in earlier publications of this study (32, 33).

### Structured Interviews

At baseline, a socio-demographic questionnaire, the Opiate Treatment Index (OTI) (34) and the Mini International Neuropsychiatric Interview (MINI) V7.0.2 for DSM-5 (35) were administered. The OTI includes sections on drug use, injecting and sexual behavior, social functioning, criminality, and general health. Each section is scored with a higher score indicating more severe dysfunction. The MINI was used to diagnose the following conditions: major depressive episode, bipolar disorder, generalized anxiety disorder, psychotic disorder, post-traumatic stress disorder, obsessive compulsive disorder, social anxiety disorder and antisocial personality disorder.

Suicidal ideation and behaviors (SIB) were assessed using the suicidality section of the MINI. The suicidality section comprises 19 questions which assess thoughts of suicide and suicide gestures during the past month (current SIB), intent to die, lifetime suicide attempts and likelihood to attempt suicide within the ensuing 3 months. Questions are individually weighted (e.g., an affirmative response to past month thoughts of wishing one was dead received one point, however an affirmative response to a likelihood of a future suicide attempt in 3 months 13 points). The final score represents the summation of all points assigned to positive responses. A score of 1–8 is rated as a low level of SIB, 9–16 as moderate and a score ≥17 as high. At both follow-up points the OTI, MINI and a follow-up interview were administered.

### Statistical Analysis

Categorical variables were summarized by frequency and percentage tabulation. Continuous variables were summarized by the mean, standard deviation (SD), median and interquartile range (IQR). To determine the impact of detoxification-based treatment on SIB, we evaluated the change in SIB scores from baseline (entry into treatment) to t1, t1 to t2 and baseline to t2. To identify the correlates of baseline and future suicide risk, the relative risk (RR) of each study variable (independent variables) for a high SIB score at baseline (outcome 1) and t2 (outcome 2) was determined together with its 95% confidence interval, using binomial regression. We focused on the outcome of a high SIB score as the participants who fell within this category likely represent those who were most at risk for suicide (MINI score ≥ 17).

The baseline variables included in the analysis comprised socio-demographic variables (age, gender, employment status, marital status, number of children, living companions and accommodation), substance history variables (age of onset of heroin use, duration of heroin use, number of substances used, individual substances used), clinical variables (HIV status, any mental illness (MI), injecting and sexual behavior score, social functioning score, general health score, criminality score and treatment-related variables (completed the program, requested early termination of treatment, dismissed from the program, absconded or transferred to another facility). The following t1 variables were included in the univariable analysis: employment status, substance use variables (individual substances used, number of substances used), clinical variables (antisocial personality disorder (ASPD), any MI (excluding ASPD), injecting and sexual behavior score, social functioning score, criminality score, general health score) and treatment variables [attended an aftercare program (including Narcotics Anonymous groups)].

Variables with an *n* ≥ 15 that showed significant univariate association with SIB were combined into a multivariable model, after examining each pair of variables for possible confounding using the chi-squared test (or Fisher's exact test for 2 x 2 tables) (36). A value of Cramer's V (or the phi coefficient for Fisher's exact test) > 0.60 was regarded as too strong an association to include both variables in a multivariable model. Non-significant variables were sequentially removed from the multivariable model. Data analysis was carried out using SAS version 9.4 for Windows (37). The 5% significance level was used.

## Results

### Enrolment and Follow-Up

A total of 304 participants signed informed consent and were enrolled in the study. During baseline interviews, four participants were found to be actively suicidal with an imminent plan and intent to attempt suicide. One of the participants had attempted suicide, by means of hanging the night prior to the study interview. As per the study protocol, all four participants were excluded from the study and referred to the treating team for urgent medical attention. The baseline sample thus comprised 300 participants, of which a full description can be found in a previous publication (32). 84% (*n* = 252) and 75% (*n* = 225) of the participants were seen at t1 and t2, respectively. Over the study period, four participants demised; three due to medical illness and one due to trauma related injury. There were no known reports of death due to suicide; however, the whereabouts of 13.7% of participants at t1 and 21% at t2 that were lost to follow-up are unknown.

At t1, there were no observed differences between those lost to follow-up vs. those still included on demographic variables, substance abuse history, psychopathology and HIV status. However, those followed up successfully had a higher proportion of participants living in formal housing at enrolment, compared with those lost to follow-up (*p* = 0.042). At t2, there were several significant differences between those seen and those lost to follow-up: those followed up successfully had a higher median duration of heroin use (7 vs. 6 years; *p* = 0.033), a lower proportion who used cannabis without heroin (*p* = 0.048), a lower proportion with mental illness (*p* = 0.047), a higher proportion of methaqualone users (*p* = 0.0098) and a higher proportion of those living in formal housing (*p* < 0.0001).

### Cohort Characteristics

At baseline, the median age at study enrolment was 27 years (y) (IQR 23–30y). The sample comprised 256 (85.3%) men and 44 women and the majority were unmarried (97.3%). A total of 167 (55.7%) participants had completed 10 to 11 years of schooling. Fifty-four percent reported some form of employment. Of those employed, 147 (90.7%) received their income from informal jobs such as recycling, guarding cars and working as street vendors. Two-hundred and three (67.7%) participants lived with their family, and 17.7% reported being homeless and living on the streets. Of those who reported testing for HIV at any point in their lifetime, 18.5% were HIV positive.

The median age of onset of heroin use was 19 years (IQR 17–22y) and the median duration of heroin use was seven years (IQR 4–9y). At baseline, 162 (54.0%) used three or more substances (other than tobacco) (Table 1). At 3-month follow-up 65.5% of those seen had continued heroin use and at 9-months 72.4% were using heroin. Further details of the substances used over the study period as well as median social functioning, and general health scores are presented in Table 1. 49.3% were diagnosed with a mental illness (MI) (excluding ASPD) at baseline. 40.9 and 37.3% were diagnosed with a MI at t1 and t2, respectively (Table 1).

**Table 1:** Clinical characteristics over 9-months.

	**Baseline (** ***n*** **−300)**		**t1 (** ***n*** **−252)**		**t2 (** ***n*** **−225)**	
	***N***	**%**	***N***	**%**	***N***	**%**
**Past month substance use**
Heroin	300	100	165	65.5	163	72.4
Cannabis	259	86.3	184	73.0	185	82.2
Crack/Cocaine	78	26.0	43	17.1	51	22.7
Crystalmetamphetamine	58	19.3	59	23.4	46	20.4
Methaqualone	55	18.3	43	17.1	47	20.9
Alcohol	49	16.3	139	55.2	103	45.8
≥3 substances used	162	54.0	128	50.8	132	58.7
Diagnosis of a mental illness (excluding ASPD)	148	49.3	103	40.9	84	37.3
ASPD	157	52.3				
Median Social functioning score (IQR)	27 (22.5–31)		23 (19–27)		23 (19–27)	
Median General health score (IQR)	7 (5–8)		4 (2–7)		6 (3–8)	
Median injecting and sexual behavior score (IQR)	5 (0–12)		3 (0–6)		4 (2–8)	
Median criminality score (IQR)	4 (1–6)		0 (0–2)		0 (0–3)	
SIB (score > 0)	^*^206	68.7	98	38.9	^**^106	47.1

* *Baseline vs. t1: p = 0.0049,*

** *t1 vs. t2: p = 0.031*.

### Treatment Received Over 9-Months

At t1 of the 252 participants followed up, 30 participants (11.9%) received some form of formal treatment post inpatient rehabilitation. These comprised attending a halfway house (*n* = 4), individual sessions with a social worker (*n* = 17), and being readmitted to inpatient detoxification (*n* = 9). 37% had attended one or more Narcotics Anonymous (NA) meetings. None were prescribed OAMT as a maintenance treatment. Of the 225 participants seen at t2, five participants were receiving intermittent OAMT prescribed by a private general practitioner and 26 participants (11.6%) reported that they were currently receiving formal psychosocial therapy.

### Suicidal Ideation and Behavior Over 9-Months

68.7% (*n* = 206) of participants at baseline endorsed SIB (any score above 0) (Table 1). The proportion of those who endorsed SIB decreased significantly to 38.9% at t1 (baseline vs. t1: *p* < 0.0001) and thereafter increased to 47.1% at t2 (t1 vs. t2: *p* = 0.047) (baseline vs. t2: *p* < 0.0001). Of those who endorsed SIB, 75.2% had high, 4.9% moderate and 19.9% low SIB scores at baseline (Table 2). At baseline, 34.0% reported a history of one or more suicide attempts in their lifetime. 8.7% of those who endorsed SIB at baseline stated that they were likely to attempt suicide in the following 3 months. The proportion of those who were likely to attempt suicide in the following 3 months increased significantly to 16.3% at t1 (baseline vs. t1: *p* = 0.0049) and 22.7% at t2 (t1 vs. t2: *p* = 0.031), (baseline vs. t2: *p* < 0.0001) (Table 2).

**Table 2:** Categories of SIB over 9-months.

	**Baseline** ***n* = 206 (%)**	**t1** ***n* = 98 (%)**	**t2** ***n* = 106**	***p*-value** **(baseline vs. t1)**	***p*-value** **(t1 vs. t2)**	***p*-value (baseline vs. t2)**
Low score (1–7)	41 (19.9)	28 (28.6)	34 (32.1)			
Moderate score (8–16)	10 (4.9)	11 (11.2)	10 (9.4)			
High score (≥17)	155 (75.2)	59 (60.2)	62 (58.5)	^*^ <0.0001	^*^0.43	^*^ <0.0001
Past month suicidality	200 (66.7)	93 (37.1)	100 (44.4)	<0.0001	0.056	<0.0001
Suicide likely in following 3 months	26 (8.7)	41 (16.3)	51 (22.7)	0.0049	0.031	<0.0001

* *Comparison between a high score and low/moderate score*.

### Correlates of a High SIB Scores at Baseline

The relative risk (RR) of each variable at baseline for a high SIB score was analyzed univariately and then in a multivariable analysis (*n* = 300). Table 3 presents the factors that were significantly associated with a high SIB score in the univariable analyses. These were: use of other opiates (beyond heroin), use of substances other than heroin and cannabis, a higher crime score, a higher general health score and diagnosis of a MI. After controlling for all other variables, in the multivariate regression model the significant independent risk factors for a high suicidality score at baseline were a diagnosis of a MI (RR 1.63; 95% CL 1.30–2.03) (*p* < 0.0001) and a higher general health score (indicative of poorer health) (RR 1.02; 95% CL 1.01–1.04) (*p* = 0.001) (Table 3).

**Table 3:** Factors significantly associated with a high SIB score at baseline.

**Univariable analysis**							
**Variable**	**SIB score at baseline**			
	**0–16**		**≥17**		***p*** **-value**	**RR for high SIB score**	
	***n***	**%**	***n***	**%**		**RR**	**95% CI for RR**
Use of opiates (other than heroin)	5	22.7	17	77.3	0.0008	1.55	1.20–2.00
Use of substances other than heroin and cannabis	71	41.0	102	59.0	0.0048	1.41	1.11–1.80
Mental illness	46	31.1	102	68.9	<0.0001	1.95	1.55–2.92
Crime Score: median (IQR) (RR is per point)	3 (1–5)		4 (2–7)		<0.0001	1.06	1.03–1.09
General health score: median (IQR) (RR is per point)	18 (13–22)		22 (17–26)		<0.0001	1.03	1.02–1.04
**Multivariable analysis**							
**Variable**					***p*** **-value**	**RR for high SIB score**	
						**RR**	**95% CL for RR**
Mental illness					<0.0001	1.63	1.30–2.03
Median general health score (RR is per point)					0.001	1.02	1.01–1.04

All variables that were tested for an association with a high SIB score at baseline are included in the Supplementary File 1.

### Correlates of High SIB Scores at t2

The RR of each variable at baseline and t1 for a high SIB score at t2 were analyzed univariately and then in a multivariable analysis (*n* = 210). The baseline and t1 variables that were significantly associated with a high SIB score at t2 in univariable analyses are presented in Table 4. In the multivariable analysis, the significant independent risk factors for a high SIB score at t2 were a diagnosis of a MI at t1 (RR 2.73; 95%CL 1.78–4.19) (*p* < 0.0001) and a higher social functioning score (indicating poorer social functioning) at baseline (RR 1.07; 95% CL 1.04–1.11) (*p* < 0.0001).

**Table 4:** Factors significantly associated with a high SIB score at t2.

**Univariable analysis**							
**Baseline variables**	**SIB score at t2**						
	**0–16**		**≥17**		***p*** **-value**	**RR for a high SIB score**	
	***n***	**%**	***n***	**%**		**RR**	**95% CI for RR**
Female gender	16	57.1	12	42.9	0.020	1.81	1.10–2.99
Unemployed	60	65.9	31	34.1	0.030	1.66	1.05–2.62
Number of children (grouped): one child	38	65.5	20	34.5	0.030	1.74	1.06–2.86
Number of children (grouped): two or more children	16	61.5	10	38.5	0.031	1.94	1.06–3.53
Homeless/living on the streets	13	50.0	13	50.0	0.0026	2.10	1.30–3.41
Age at first substance use: mean (SD) (RR is per year of age)	14.7 (2.8)		14.3 (2.5)		<0.0001	0.84	0.78–0.90
Past month alcohol use	20	57.1	15	42.9	0.0087	1.88	1.17–3.00
Social functioning score: median (IQR) (RR is per point)	26 (22–29)		27 (22–31)		<0.0001	1.10	1.06–1.13
Crime score: median (IQR) (RR is per point)	3 (1–5)		4 (2–7)		0.021	1.07	1.01–1.14
General health score: median (IQR) (RR is per point)	19 (14–24)		24 (22–30)		<0.0001	1.07	1.04–1.11
Mental illness (excl. ASPD)	63	67.0	31	33.0	0.046	1.59	1.01–2.52
**T1 Variables**							
Past month heroin use	92	68.7	42	31.3	0.032	1.83	1.05–3.19
Past month alcohol use	95	79.8	24	20.0	0.025	0.59	0.37–0.94
Past month crack/cocaine use	22	59.5	15	40.5	0.021	1.75	1.09–2.82
Mental Illness	43	52.4	39	47.6	<0.0001	3.80	2.28–6.35
Injecting and Sexual behavior score: median (IQR) (RR is per point)	4 (2–7)		4 (0–7)		0.0029	1.04	1.01–1.06
Social Functioning score: median (IQR) (RR is per point)	22 (18–25)		23 (19–28)		<0.0001	1.10	1.06–1.13
General Health score: median (IQR) (RR is per point)	4 (4–17)		14 (5–22)		<0.0001	1.05	1.03–1.07
Crime score: median (IQR) (RR is per point)	0 (0–1)		0 (0–3)		0.0069	1.09	1.02–1.16
**Multivariable analysis**							
**Variable**					***p*** **-value**	**RR for high t2 SIB score**	
						**RR**	**95% CL for R**
Mental illness at t1					<0.0001	2.73	1.78–4.19
Baseline social functioning score: median (IQR)(RR is per point)					<0.0001	1.07	1.04–1.11

All baseline and t1 variables that tested for an association with a high SIB score at t2 can be found in the Supplementary File 1.

## Discussion

This is the first study to longitudinally investigate the patterns and correlates of suicidal ideation and behavior (SIB) in a cohort of heroin users from Sub-Saharan Africa. The results demonstrate that a significantly large proportion of the cohort endorsed SIB at all three timepoints, the majority of whom fell within the category of a high SIB score. Studies from high income regions report a SIB prevalence of 22.5%-33% among heroin users at baseline (9–11). In comparison, our study findings show that 68.7% endorsed SIB at baseline. Concerningly, after inpatient detoxification and psychosocial rehabilitation there was a significant increase in the proportion of those reporting a likelihood of suicide in the ensuing 3 months. Also, from 3- to 9-month follow-up there were observed increases in the proportion of those reporting any SIB. We frame our discussion to address two key questions: why were SIB scores approximately 3 times higher in our LAMIC sample compared to data from high income countries? Why did SIB remain relatively high throughout the study period, even though the cohort had engaged with a treatment service?

In our cohort, 66.7% reported past month SIB at baseline and 44.4 % at 9-month follow-up. In comparison, among a cohort of 387 heroin users from the Australian Treatment Outcome Study (ATOS), 22.5% reported current suicidal ideation at baseline and this dropped significantly to 6.7% at 12- month follow-up (9). There were no substantial differences between the South African cohort and the Australian cohort in terms of average age of participants (27 vs. 29.4 y), years of education (10–11 vs. 10.2 y), age of onset of heroin use (19 vs. 18y) and length of heroin use (7 y in both cohorts). We contextualize our findings and the treatment system in South Africa to understand and make comparisons to other studies in the field, such as ATOS.

Different from the treatment priority in some high-income countries, opioid agonist maintenance therapy (OAMT) is not a nation-wide or standard treatment option for heroin users in South Africa (38). Our study cohort received detoxification-based treatment. As reported in a previous publication (32), most state-funded substance rehabilitation facilities in South Africa are nested within the Department of Social Development. Therefore, they often cannot comprehensively manage all psychiatric and non-psychiatric comorbidities at the treatment site. This was evidenced in the afore mentioned paper where despite almost 50% being diagnosed with a MI (in research interviews) just three of the 252 participants seen at follow-up were on psychotropic medication and of those, two had seen a psychiatrist. Comparatively, in the Australian cohort 74.2% were exposed to OAMT over a 36 month period and 31.2 % of the cohort were on antidepressant medication (9, 39). The limited access to assessment and treatment of psychiatric comorbidities as well as the lack of OAMT in our study may explain some of the differences between current suicidal ideation in the Australian and South African samples. In our sample, attendance at psychosocial aftercare treatment (including Narcotics Anonymous groups) did not significantly decrease the risk of SIB.

Increases in the proportion of participants who reported a likelihood of suicide in the following three months may be related to feelings of hopelessness in participants who had continued to use heroin post detoxification. The increase in SIB from t1 to t2 may further indicate a trend toward a return to baseline levels of SIB and highlights the non-sustainability of an only detoxification-based treatment approach. Importantly, Taiwanese and Danish national data base studies evaluating the predictors of death by suicide in substance users found that a comorbid MI was significantly associated with mortality. Both studies concluded that screening and treatment for comorbid MI in substance users is necessary to prevent suicide (7, 19).

One of the challenges when comparing suicide data is the range of definitions used for suicidality, suicidal behavior and suicide attempts. The suicide scale within the MINI lends itself to the broad term of SIB used in this study. Those with high SIB scores likely represent the proportion of the cohort that was most at risk for suicide and therefore we wanted to identify predictors of future SIB. After controlling for all other variables, poorer general health and a diagnosis of a MI at baseline was associated with a high SIB score at baseline. A diagnosis of a MI at 3-months and poorer social functioning at baseline was associated with a high SIB score at 9-months. These findings were expected and in line with previous studies where comorbid MI (6, 17, 22), social isolation (9), and poorer physical health (17, 22) were significantly associated with suicide attempts in heroin users.

However, some variables reported in other cohorts such as female gender (6, 17) and number of classes of substances used (9, 22, 29, 38) were not significantly associated with SIB in the current study. A possible reason for differing findings may be that the outcome assessed in this study was SIB, compared to suicide attempts in previous studies. The low number of female participants and reliance on self-report alone, when MDUTs were not available, is a limitation of the study. Additionally, the study was unable to account for the specific impact of intoxication and withdrawal symptoms on SIB. Importantly, the results point to a cohort who had significant risks for SIB and one that was highly vulnerable to suicide. However, no specific suicide screening and preventative intervention was offered to the cohort at any timepoint.

The 2020 publication of the International Standards for Treatment of Drug Use Disorders (40) states the need for the incorporation of suicide screening for substance users at all levels of assessment. The same document also recognizes the fragmentation of services for people with substance use disorder and suggests a “one stop shop” organizational model where a wide range of medical and social services are provided in one facility (41). Where this is not easily achievable, social, mental health and general health services should work toward strengthening coordination and communication and provide low-threshold community-based entry points for service users (40). All these measures require much needed policy reform, planning and funding at national and provincial levels. The US based Substance Abuse and Mental Health Services Administration (SAMHSA) released a brief which describes a few practical steps for managing the nexus of suicide and substance use. Some of these include provision of information to substance use professionals about the risk of suicide among their patients, training of substance use professionals to evaluate suicide risk and for substance use professionals and suicide prevention professionals to familiarize themselves with local referral and treatment programmes (42).

In regard to suicide screening tools, Boudreaux and Horowitz (43) suggest the development of tools that take into account specific parameters related to each setting such as the infrastructure available and the scope of the practice. It is highly recommended that suicide screening tools inform actionable clinical risk. Additionally, tools should be vigorously validated in an array of settings. Although a few suicide screening tools such as the Ask Suicide-Screening Questions (ASQ) (44) and the Columbia–Suicide Severity Rating Scale (45) can be used in clinical settings, their use in a substance using population has not been evaluated. Given the high rate of suicide in LAMIC, and among substance users, it is important to consider development of suicide screening tools for use in these particular settings (1, 30).

## Conclusion

The results of this study suggests that suicide behaviors and thoughts persist among heroin users after detoxification. Suicide screening tools that are tailored for use in LAMIC clinical settings and for people with SUD are needed. Our data supports substance treatment programs that include suicide screening at all points of contact and takes into account comorbid mental and physical illnesses. In order to provide these services, high level coordination and communication between health and social sectors are urgently needed. Directing resources toward evidenced-based treatment for the highly vulnerable population group of heroin users will more adequately tackle the public health issue of suicide.

## Data Availability Statement

The original contributions presented in the study are included in the article/Supplementary Material, further inquiries can be directed to the corresponding author.

## Ethics Statement

The studies involving human participants were reviewed and approved by Human Research Ethics Committee (Medical)- University of the Witwatersrand. The patients/participants provided their written informed consent to participate in this study.

## Author Contributions

NM collected the data and wrote the first draft of the manuscript. All authors contributed to the study design, interpretation of the data, and contributed to subsequent revisions of the manuscript.

## Funding

This study was supported by the Alexander von Humboldt Foundation, the M&J Miller Foundation and the South African National Research Foundation (TTK170430229217).

## Conflict of Interest

The authors declare that the research was conducted in the absence of any commercial or financial relationships that could be construed as a potential conflict of interest.

## Publisher's Note

All claims expressed in this article are solely those of the authors and do not necessarily represent those of their affiliated organizations, or those of the publisher, the editors and the reviewers. Any product that may be evaluated in this article, or claim that may be made by its manufacturer, is not guaranteed or endorsed by the publisher.

## Footnotes

^1^The ratio of observed deaths in the study group to expected deaths in the general population.

^2^For the purposes of this document, suicidal ideation refers to thoughts of suicide; suicidal behavior includes planning a suicide as well as non-fatal suicide attempts and suicide refers to the deliberate act of killing oneself.
